# Æsthetic Dentistry

**Published:** 1883-11

**Authors:** E. H. Bunting

**Affiliations:** Newark, N. J.


### SELECTIONS.

### Æsthetic Dentistry.

BY DR. E. H. BUNTING, NEWARK, N. J.

A pleasing feature of the nineteenth century progress is the tendency of modern artists to treat all subjects from an æsthetic as well as a sanitary stand-point.

Health and beauty are synonymous, and science was never so potent a sovereign as in the present methods vouchsafed for their preservation. As an adjunct to physical well-being, the teeth are all-important. Health and life depend upon the aid they render in moving the intricate machinery of the human system. They are the pivots upon which many forces turn.

"No man is a man minus his molars," is scarcely an exaggerated statement of the state to which the "lord of creation" is reduced by the absence of teeth. Digestion is retarded and he becomes a prey to nightmares innumerable. Life loses its savor if deprived of those pleasures of the palate which caused the Sybarite to desire a lease of his life for a thousand years.

The slightest failure upon the part of these diminutive accessories to perform their functions will clog the ponderous wheels of toil, and strike discord from the most intricate mental or moral machinery; in short the scientist was not far wrong who likened any derangement of the delicate nerves which find lodgment in the teeth to a subtle demon who plays Delilah to the veriest Samson in our midst.

So much for results to which we are indebted for some of the grandest triumphs of modern science.

The annals carry us a long way backward to the master whose handiwork has swayed constructive artists to whom even Mozart's majesty was a myth, from the laboratory to the sanctuary. Here we bow, abashed by an original triumph which modern handicraft may never attain.

Now, concerning this beatific construction, let us inquire. Every detail is masterful. There is no halting in the grand economy of forces forming the whole. The form is symmetrical as that of the Venus de Medici, the chevalure and complexion perfect as that for which the fair Castilian was famed—the eyes are of course divine, and the teeth "Eugenia." Now we come to the point at issue.

The mother being the first minister, and the first artisan of humanity, her first care should be the transmission of pure blood and sound muscle to her progeny. She should think the thoughts that will make them strong, and eat the food which will render them vigorous and enduring. Every child has a divine right to be beautiful, and if it be not so there is an omission somewhere. If the supple limbs be unshapely, then must art come to the rescue and perform what nature under pressure has failed to achieve. If the spine fails to fulfill its functions, she resorts to the electric stimulus which supply lack of strength and vigor. If the complexion be sallow and lacking in the tints essential to beauty, we must look to the diet, selecting as a matter of constructive economy certain flesh-forming substances which will best clear the muddle. If the eyes be weak, those ministrations which at once strengthen and invigorate the brain must be sought, all the time remembering that bone and sinew have each its mission in performing the work of the system, as well as contributing to the external perfection of things which betray the infinite origin.

There is not in the annals of all the creations a construction which can compare to the human frame. The first attempt at critical analysis of its composing elements will go very far toward convincing even a skeptic of the divinity of the human species. Every atom is perfect of itself; and yet the absence of the smallest one would mar the harmonious action of the whole.

There is, perhaps, no avocation on earth where so much is left to conscience as in the matter of treating the mouth whose teeth have been left to take care of themselves until nature has broken out into an unquenchable revolt. Pain—and such pain—is an argument which humanity is slow to resist. The strongest constitution is swayed as a reed in high winds, by the iron grip of an agony for which there is no resource save the formidable forceps, of which humanity has a wholesome dread. The mission of the dentist is to remove pain by first inflicting pain! Par consequence, we hold him in that sort of blood-curdling awe with which we regard the ghosts of duties unperformed.

These issues constitute a fertile field for the exercise of that foresight which would prove to modern science an invaluable aid.

Again let us retrace our steps. The old adage "Two steps backward to one forward" is more clearly demonstrated in the annals of dentistry than, perhaps, in any other science. Mothers should look especially to the formation of the teeth of their offspring. Certain foods tend to the production of strong teeth which will not crumble at the touch of sweets, or discolor under the influence of modern esculents. Teeth that betray the work of the cereals in their construction; teeth that will do to live by and to die by; teeth which are suggestive of the strength, vigor, and character they are destined to express, are an adjunct as essential to the perfection and harmony of the human figure as the eyes, which are mirrors of the divine, or the flesh, typical of humanity.

These accessories of the system are of slow and painful growth. They are, in truth, a second birth born of the necessities of digestion, and they are, as a general thing, sufficient, not only as an element of beauty, but an indispensable appendix to health. If we could examine at will the death record, we should with few exceptions find that the diseases which proved fatal had their origin in lack of mastication resulting from imperfect or insufficient teeth. If people only realized the importance which attaches itself perforce to this branch of constructive economy, the dentist's chair would cease to have the terrors which now menace the victims of imperfect teeth.

They should be good and beautiful, for nature is a royal benefactor, who never fails of herself. Her resources, if not weakened by use or impaired by disease, are simply perfect. The necessities of approximating, as nearly as may be, the original standard, have imparted an æsthetic side to this sensitive subject.

The conscientious mother will be a scrupulous guardian of the texture, shape, and growth of the teeth of her charge. She will esteem her dentist an incident or an accessory to household economy quite as important as the minister or family doctor, to whom she flies under pressure of those ills to which all flesh especially infant flesh, is heir. Never was the old adage concerning that ever seasonable and potent "ounce of prevention" more forcibly exemplified than in the treatment of the teeth. There are, of course, instances in which all precautions fail. The enemy will come, despite the vigilance which does not slumber at its post.

The richest and most varied resources of nature must fail anon, and she avail herself of the immunities of her handmaiden, art.

Now we come to the developments of modern dentistry, which embody an art so perfect as to conceal even the trace of art. Let us reverently enter the domain in which the genius of the present practitioner reigns supreme.

The instant the patient is in the chair, with that sublime instinct to repair damage and to remove pain, comes the perception which is half an inspiration, and which enables him to decide concerning the tastes and habits of his subject, and to adopt the remedies at hand.

To supply, in any sense, the deficiency of nature, is a grand thing. The artist-dentist will, of course, avoid all appearance of the craft which savors of supply. If there be pain the first duty will be to remove the cause; this done, of course, study the effect, keeping nature, the grand first cause, perpetually in view.

The artist-dentist will study the requirements of the mouth with which he has to deal as carefully as does the physician the system in which his remedies are expected to restore health and perpetuate life. A knowledge of the tone and habits of the pa ient are issues of vital interest upon which, oft-times, a cure depends. Like the dentist, he too finds it essential to pull down before building. It is poor policy to rebuild a structure upon an unsound basis. Decaying sleepers and crumbling masonry if not removed, must be so thoroughly repaired that not a vestige of the spoiler remains.

The necessity of making a clean mouth to begin with bears upon its face the fallacy of a certain tooth-crowning process which may prove an excellent adjunct in improving or altering the appearance of the mouth if applied to sound teeth, that is, if the accessory be fitted so accurately as to prevent the lodgment of food particles, which, the crowns being stationary, are not easy to remove.

This we esteem an innovation on established laws, a trifle incomplete, to say the least. Other departures are more decided and prolific of results pleasing and profitable.

It is, of course, no part of my object to discourse upon the science so familiar to my learned colleagues of the chair; yet one fact suggests itself as worthy of repetition.

The natives of Northern Siberia have singularly healthy teeth. Old men of sixty or seventy have sets of teeth small and pearly white, polished and healthy. Decay and suffering are unknown. A physician of Yakiotake attributed this to their habits, and the kind of food eaten by the natives, and to a certain care taken by them from childhood up. First, the natives do not touch sugar in any form, for the simple reason that they cannot afford to buy it. Secondly, they are in the habit of drinking large quantities of fermented sour milk, summer and winter, which is antiscorbutic, and is very beneficial in preserving the teeth. And lastly, they have the habit of chewing a preparation of the resin of the fir tree, a piece of which, tasting like tar, they masticate after each meal, in order to clean the teeth and gums of particles of food that may remain after eating. The gum, or resin, is prepared and sold by all the apothecaries in Siberia, and is much used by Parisian ladies.

A marked triumph of modern dentistry has been to take from false teeth the ghastly aspect imparted by an unnatural whiteness; a wholesome tint is imparted and an assumption of naturalness observed which would readily baffle the perception of critics unfamiliar with the tricks of the craft. Modern artists are careful to avoid any appearance of setness in the formation. Many times a facial defect may be remedied in the second set of teeth.

The old tragedy of Fantine selling her beautiful teeth, as told so touchingly by Victor Hugo in "Les Miserables," recurs less frequently, though now and then the demand for transplanted teeth is successfully dealt with. If the tiniest cavity can be found, the filling will increase the appearance of naturalness, which is the acme of art.—*The Independent Practitioner.*

